# Supplementary figures and images for: Micro-dissection and integration of long and short reads to create a robust catalog of kidney compartment-specific isoforms
Source: PLoS Comput Biol. 2022 Apr 25;18(4):e1010040. doi: 10.1371/journal.pcbi.1010040 (PMC9037928; doi:10.1371/journal.pcbi.1010040)

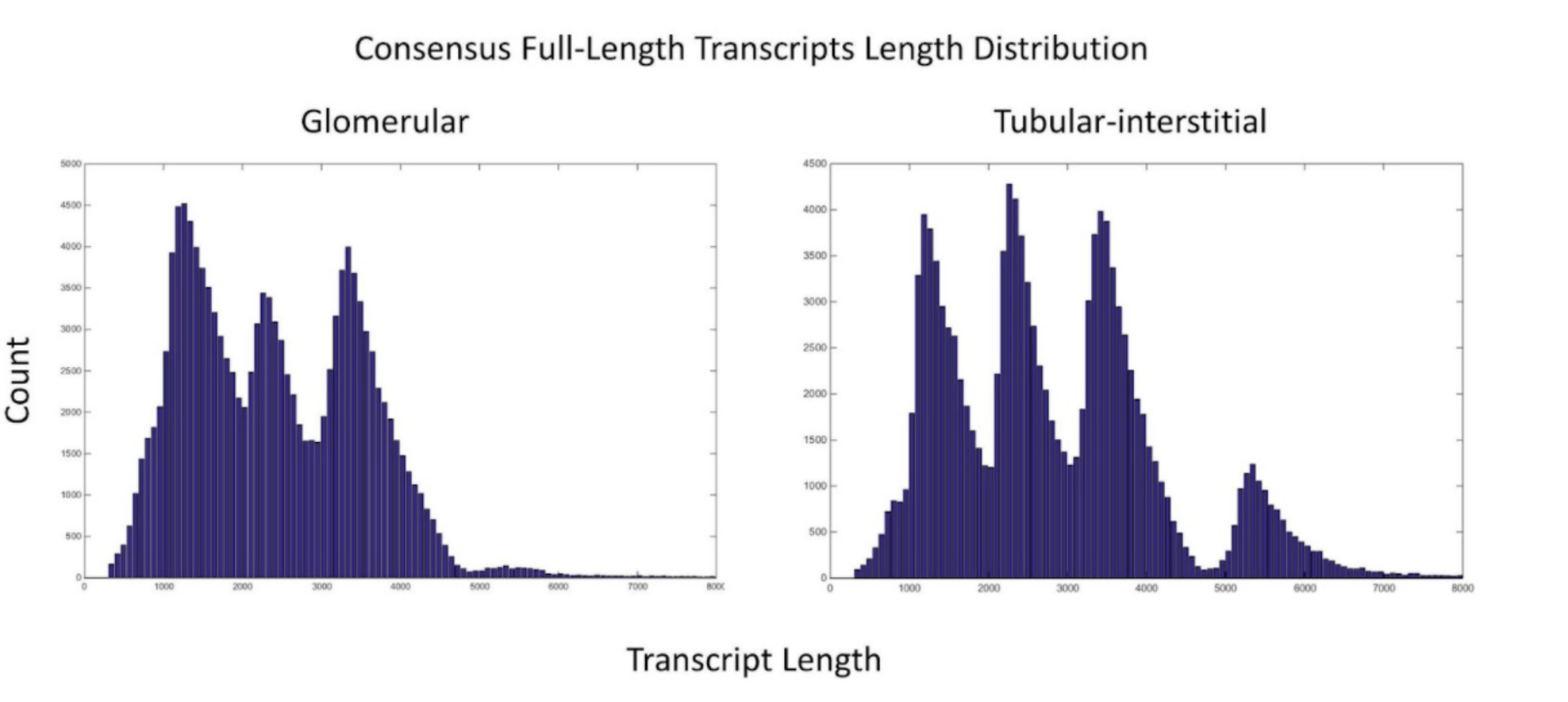

Supplement: S1 Fig — (TIF) [file pcbi.1010040.s001.tif]
