# Supplementary material for: Micro-dissection and integration of long and short reads to create a robust catalog of kidney compartment-specific isoforms
Source: PLoS Comput Biol. 2022 Apr 25;18(4):e1010040. doi: 10.1371/journal.pcbi.1010040 (PMC9037928; doi:10.1371/journal.pcbi.1010040)
Supplement: Attachment — Submitted filename: response.pdf [file pcbi.1010040.s003.pdf]

Reviewer #1: In this paper, the authors used both PacBio Iso-Seq long reads and Illumina RNA-seq short reads' information to analyze and identify expressed isoforms for defining microscopic structures. They introduced a pipeline, in which they used long-reads of kidney tissues from two compartments: glomerular and tubule-interstitial and validated the reads by RNA-seq reads of the same tissue. From their experiment, they identified a substantial number of transcription start sites (TSSs), transcription end sites (TESs) and Splice junctions from a filtered set of high quality transcripts.

The manuscript is well written and easy to understand. The figures and tables are also clear and elaborate. However, I have some concerns about the work:

Thanks for your time in reviewing this manuscript. Please see our response below.

Major:

1. The novelty of the study is limited. The authors use the existing methods to analyze the Iso-seq and RNA-seq data, and identify expressed annotated and unannotated isoforms. The contribution to the computational biology field is not clear. This study can be easy applied to other tissue samples with both matched RNA-seq and Iso-seq data.

The main novelty of this study lies in an important/gold standard data resource generated for the kidney computational research. Usually, it is very challenging to obtain such biopsy and microdissection data. To reach the dataset we presented in this study, it took us more than 10 years of collection of the tissues. We carried out and integrated both long and short RNA-seq reads across individuals to validate reported isoforms and discover new isoforms in two kidney compartments. These results are crucial for future kidney studies. This process is actually not easily repeated for another tissue. We feel it is an excellent example combining both computation and experiment works, and thus a good candidate for PLoS Computational Biology which likes an integral of the two.

But we do feel the above point wasn't made crystal in the original manuscript, and have now emphasized in the introduction (Page 2):

"With this data collection, we identified a large number of novel transcript isoforms and pathways in these two kidney compartments, creating an important resource for the kidney research community."

And abstract (Page 1):

"We identified distinct pathways involved in glomerular and tubulo-interstitial compartments at the isoform level, creating an important experimental and computational resource for the kidney research community."

2. hg19 annotation is quite old (2009). It would be better to run the experiments based on the latest hg38 annotation.

Hg38 was not released until late 2013. As mentioned above, the collection of the tissues (as patients do not just pop out and donate a kidney every day), and sequencing lasted over 10 years. We used hg19 as the reference genome to begin with and had many validations and back-forth checks.

Compared to the latest hg38, one majorchangeof hg38 is the annotation of centromere regions. To the best of our knowledge, most transcripts and isoforms are located outside centromere regions. We expect that our conclusions will not significantly change if all were on hg38.

Minor:

1. Abstract line 2: insterstitial -> interstitial

2. Abstract line 5: microdissed -> microdissected?

Thank you, all have been corrected.

Reviewer #2: The paper titled "Micro-dissection and integration of long and short reads to create a robust catalog of kidney compartment-specific isoforms" describes an developed experimental and computational pipeline for identifying isoforms at microscopic structure-level. It applies Pacific Biosciences SMRT analysis software and Illumina reads approach to discover novel transcripts. Although it is a promising approach, current manuscript lacks details and interpretations.

1. The authors claimed that they developed the approach for isoform identification. However, as far as I know, there are some additional methods that have been developed recent years, such as Mandalorion (Byrne et al. Nat. Comm. 2017) FLAIR (Tang et al, Nat. Comm. 2020), TALON (Wyman et al. biorxiv). I do not mean that they need to compare to all existing methods, but it is important that a comparison is performed to demonstrate the performance of this developed approach.

Thanks for pointing out the relevant works. All have now been discussed. Although available tools are often developed for different purposes such as using long reads only, comparison of pipelines is not a focus of this paper. How to integrate both long and short RNA-seq reads across individuals to validate reported isoforms and discover new isoforms across compartments of a single tissue is the focus. Previous methods are not well-suited for our situation, where compartment-specific long and short reads from multiple individuals are available. In fact, it is not that easy or routine to identify a tissue that can clearly microdissect and collect enough materials for each compartment for sequencing. We develop this unique pipeline so that it can exploit all available data from two cohorts, robustly identify known isoforms from three sources (long reads, short reads and GENCODE annotations), as well as detect potentially novel isoforms. This has been discussed more in the introduction section (Page 2).

"In recent years, many computational methods have been developed to identify isoforms through analyzing RNA-seq reads. For example, Mandalorion was developed to analyze long reads and identify isoforms at the single-cell level in murine B cells [17]. Many expressed genes, including cell surface receptors, were found to display complex isoforms. Later on, FLAIR was developed to analyze full-length transcripts, and study differential splicing events and isoforms in leukemia samples with and without SF3B1 mutation [18]. This work demonstrates the power of full-length isoform analysis in connecting different alternative splicing events in cancer. Recently, TALON was developed as a step of the ENCODE4 pipeline in analyzing long-read transcriptomes [19]. TALON was used in multiple transcriptomes to identify both known and novel transcripts across datasets and platforms."

2. In general, it would be better to describe with more details of read correction, transcript assembly, and transcript quantification in the results part of the manuscript to illustrate the power of this approach and the reason that this approach performs good, such as what are the advantages and what are the drawback of this approach.

Thank you very much for the suggestion. We have added more data analysis details to the Results section.

Page 6:

"For the Illumina RNA-seq short reads, we followed TapStation's standard protocols to assess RNA quality. We checked the quality of the final cDNA libraries using Kapa's library quantification kit following the manufacturer's recommended protocols. Then the short reads were mapped to the human

genome (hg19) using STAR version 2.5 in a 2-pass mode following the standard "ENCODE options" in the STAR manual. For the PacBio long reads, PacBio's Iso-seq protocol was used for library preparation and long-read sequencing, and PacBio's SMRT Portal was used for the initial data analyses. We processed them using SMRT version 2.3.0 and generated reads of inserts using the default parameters of polymerase full passes. The consensus full-length transcripts (CFLs) were also mapped to hg19 using GMAP. About 98% of the CFLs were uniquely mapped for both kidney apartments."

3. To test the accuracy of this approach, it would be better to provide the rate of false discovered isoforms and illustrate the reason of these false discovered isoforms.

Typically, false discovery rate is calculated when a gold standard is available, or a pseudo-gold standard is available. For example, if we only have long reads, what is the percentage of predicted isoforms that are not confirmed by junction-wise short reads? However, our approach already integrated two sources of evidence, and as a matter of fact, no prior gold standard is available. Thus, it is impossible to infer the false discovery rate.

4. For enrichment results, it would be better to show P-values, gene/transcript count for each pathway, and top pathways in one Figure.

We have updated Figure 4b by adding gene/transcript counts, P-values for the top pathways in two kidney compartments.

b.

	KEGG pathway	Count	p-value
Glomerular	Rap1 signaling pathway	77	2.66e-6
	Ribosome	54	7.85e-6
	Thyroid hormone signaling pathway	45	7.63e-5
Tubular-interstitial	Metabolic pathway	316	3.00e-7
	Ubiquitin mediated proteolysis	48	9.58e-5
	Aldosterone-regulated sodium reabsorption	19	2.65e-4

Figure 4b

5. The Introduction and Discussion sections are not comprehensive and do not present readers a view of the field. The authors should expand it and describe what are already available and what are the specific features of existing methods for PacBio data. Some recently published methods on novel isoform discovery are not cited. While I understand that this paper focuses on application of PacBio data, it is still important to summarize state-of-the-art methods to present a comprehensive view of the current state of art.

Thank you for the suggestion. We have added recent methods to the Introduction and Discussion.

Page 2

“In recent years, many computational methods have been developed to identify isoforms through analyzing RNA-seq reads. For example, Mandalorion was developed to analyze long reads and identify isoforms at the single-cell level in murine B cells [17]. Many expressed genes, including cell surface receptors, were found to display complex isoforms. Later on, FLAIR was developed to analyze full-length transcripts, and study differential splicing events and isoforms in leukemia samples with and without SF3B1 mutation [18]. This work demonstrates the power of full-length isoform analysis in connecting different alternative splicing events in cancer. Recently, TALON was developed as a step of the ENCODE4 pipeline in analyzing long-read transcriptomes [19]. TALON was used in multiple transcriptomes to identify both known and novel transcripts across datasets and platforms. ”

Page 8

“Recent advances in computational methods have shown great promise to unveil biological insights underlying the complex alternative splicing events and isoforms in a variety of cell types and tissues, such as Mandalorion [17], FLAIR [18] and TALON [19]. To address the specific situation in our work, we developed a unique in-house pipeline to integrate both long and short RNA-seq reads across individuals. This pipeline enables us to validate reported isoforms, as well as discover new isoforms in two kidney compartments.”

6. The font size in Figures is too small to read

All Fonts have been made bigger:

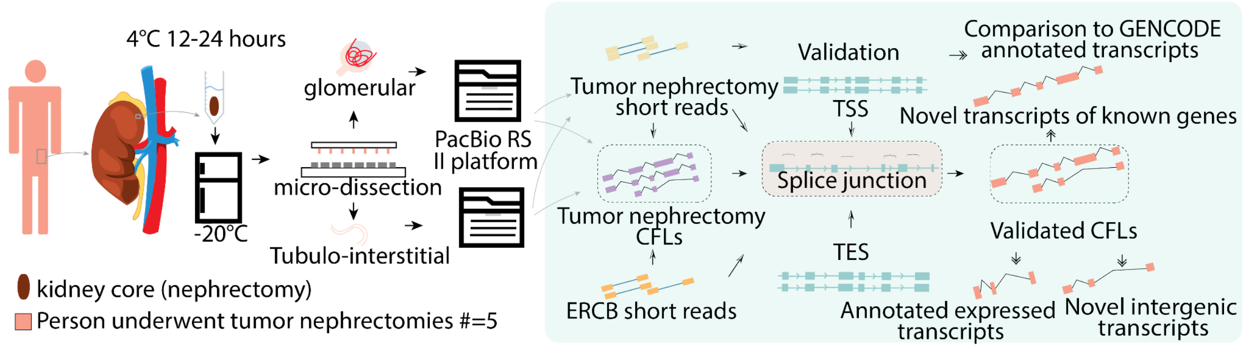

Figure 1

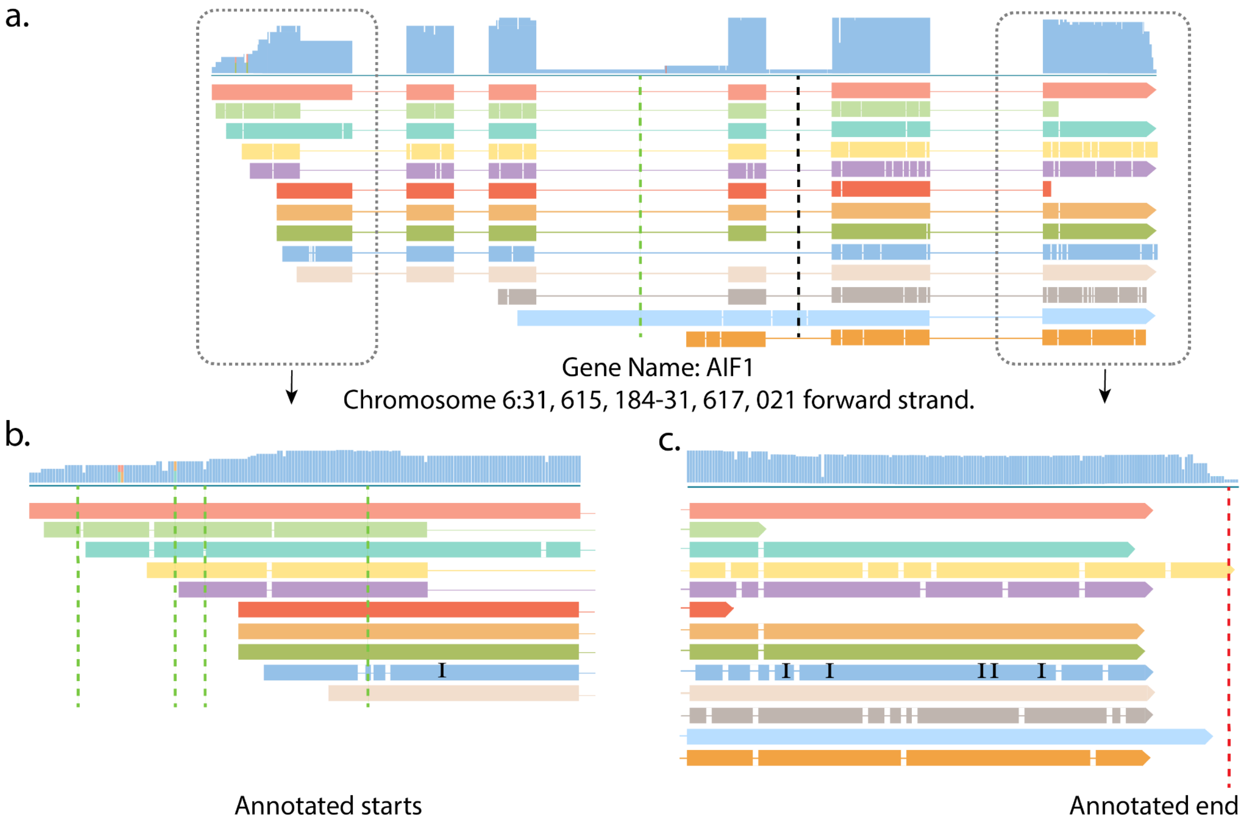

Figure 2

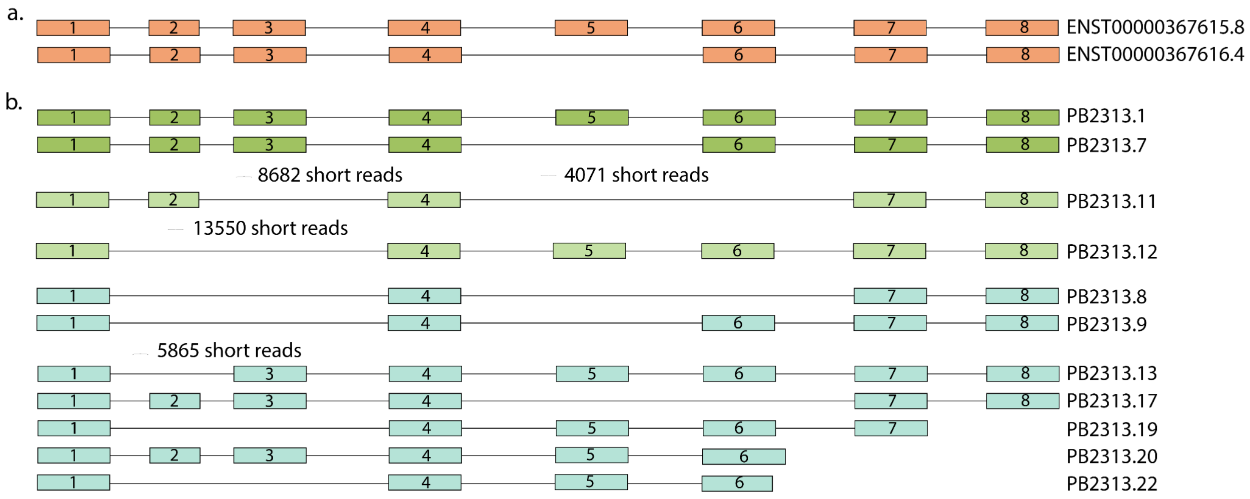

Figure 3

a.

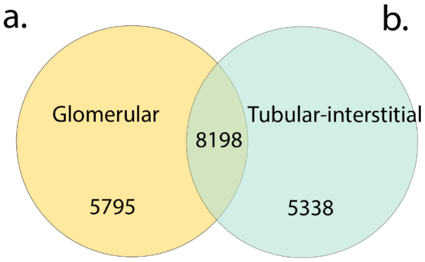

b.

	KEGG pathway	Count	p-value
Glomerular	Rap1 signaling pathway	77	2.66e-6
	Ribosome	54	7.85e-6
	Thyroid hormone signaling pathway	45	7.63e-5
Tubular-interstitial	Metabolic pathway	316	3.00e-7
	Ubiquitin mediated proteolysis	48	9.58e-5
	Aldosterone-regulated sodium reabsorption	19	2.65e-4

c.

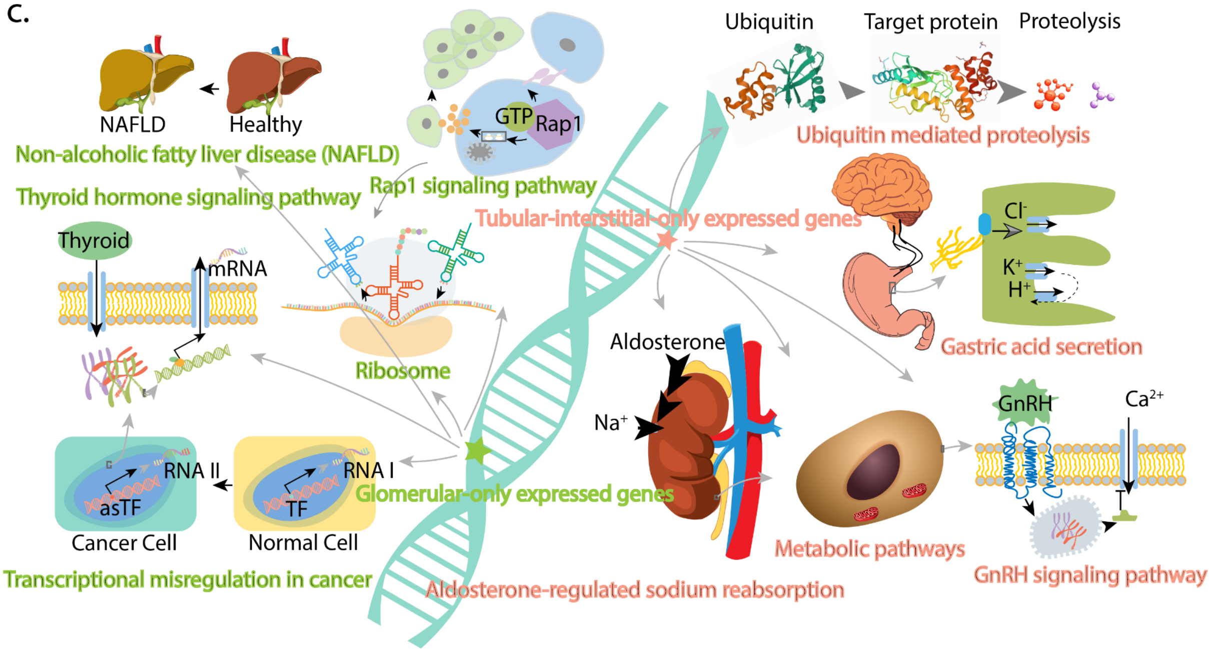

Figure 4
